# How much do we know about seabird bycatch in pelagic longline fisheries? A simulation study on the potential bias caused by the usually unobserved portion of seabird bycatch

**DOI:** 10.1371/journal.pone.0220797

**Published:** 2019-08-05

**Authors:** Can Zhou, Yan Jiao, Joan Browder

**Affiliations:** 1 Department of Fish and Wildlife Conservation, Virginia Polytechnic Institute and State University, Blacksburg, Virginia, United States of America; 2 Southeast Fisheries Science Center, NOAA National Marine Fisheries Service, Miami, Florida, United States of America; CSIRO Townsville Australian Tropical Sciences and Innovation Precinct, AUSTRALIA

## Abstract

Not much is known about the fleet level total seabird bycatch from pelagic longlines of United States vessels in the western North Atlantic or other fleets of the Atlantic or other oceans. Onboard observers generally only record seabird bycatch during line hauling. Seabirds are predominantly caught during the line setting stage, and, due to predation or mechanical action, those caught prior to the haul can drop off the hook and be lost to the onboard observer. We developed a model to gauge the size of this bycatch loss problem and provide a first approximation of its impact on estimates of total fleet bycatch. We started with a traditional loss-free bycatch model, which assumes that birds recorded were the only birds captured, and integrated into it two crucial components of the bycatch process: capture origin (set or haul) and bycatch loss of set-captures. We extracted count data on seabird bycatch loss and bycatch mortality from the literature on other longline fisheries and used these data to simulate potential total seabird bycatch in the western North Atlantic. Simulations revealed the shortcomings of both the traditional bycatch model and the current haul-only observer protocol, each of which contributed to biologically significant underestimation of total bycatch and estimation uncertainty. Based on our results, we recommend a loss-corrected modeling approach to provide a more accurate estimate of seabird mortalities in pelagic longline fisheries. Where possible, fishery-specific seabird bycatch loss rates need to be ascertained via specific set and haul observing protocols. But, even where fishery-specific estimates for a region are not available, the methodology developed here is applicable to other pelagic longline fisheries to approximate fleet-level loss-corrected bycatch.

## Introduction

On the global scale, longline fishery bycatch is a major threat to seabird biodiversity; at least 160,000 seabirds are removed by longline fishing operations annually [1]. Fishery bycatch at least partially contributed to the decline of some albatross populations on some Pacific islands [2–5]. Species diversity and the number of rare or threatened species are often high in areas of high biological production, for example, on the outer shelf off the United States Atlantic coast near Cape Hatteras [6], which is within the effort footprint of the U.S. pelagic longline fleet. Observation of bycatch when the line is hauled back to the vessel, although used by almost all pelagic longline observer programs, is not adequate to document the overall seabird bycatch because substantial bycatch loss can occur during the set but be lost before it can be observed and recorded; some caught birds escape but might subsequently die of injury [3, 7–9]. This is especially true for birds caught when the line was being set, which would go through a soak period of several hours. A long-term study in other oceans documented that 52% of seabirds caught at the line-setting stage were lost because they fell off the hook before the line could be hauled back to the vessel and the bycatch recorded [7]. With the loss rate reported by Brothers et al. [7], which included observations of set-caught birds, the ones most likely to fall off the line and not recorded by observers, the global seabird bycatch potentially well exceeds the current estimate[1].

To understand how bycatch can be lost, we review mechanisms by which seabirds can be caught but not observed by onboard observers. Many seabirds employ scavenging entirely or partially, with surface or diving feeding behavior, and such behaviors make them vulnerable to longline fishing [10–13]. The interactions of seabirds and longline operations predominantly occur at the line setting stage while baited hooks are floating on the sea surface or a few meters below the surface but still within the diving distance of some species, and also occur, but to a lesser degree, at the hauling stage when the line containing still-baited hooks is pulled near to the surface [14]. Seabirds caught during the line setting stage could drop off the hook due to predation or mechanical action [7, 8]. In addition, crew may cut off branchlines holding captured seabirds during the hauling operation, and the observer would fail to record such bycatch [15]. Birds also can be caught while the hooks are “soaking” and then drop off during the haul, like line-setting catches.

The seabird interaction observation methodology established by Brothers [3] was crucial in quantifying bycatch loss. In the following, we give an overview of that methodology, and a detailed description can be found in Gilman et al. [9]. The methodology includes both line setting and hauling observations. During line setting, observation of seabird interactions is kept for each hook after it leaves the vessel for at least 30 seconds. This requires considerable concentration and focus on the part of the observer because, for example, on average, a longline set from the U.S. western North Atlantic PLL typically consists of more than 700 hooks.

Observation of seabird-fishery interactions to this detail requires special training and adds an extra load on the observer; to the best of our knowledge, its adoption in other longline fisheries has not occurred, e.g., the current Pelagic Observer Program (POP) observation protocol does not include such a component [16]. Night-setting makes the task even more difficult. The long-term commitment required to collect sufficient data for meaningful results at the existing low encounter rate is another hindrance for obtaining more accurate or less biased estimation. Such seabird interaction observations are extremely crucial for the estimation of total direct fishery impacts on seabird populations, because they enable us to estimate a region-specific bycatch loss rate. The bycatch loss rate is an important aspect of the seabird bycatch process without which fleet-level bycatch totals are substantially under-estimated. Without a bycatch loss component, the estimated total seabird bycatch from a traditional loss-free assessment model would be only the observable fraction of the bycatch at the hauling stage. Uncertainty in the classification of the seabird-fishery interaction is another concern; it was not incorporated into the original analysis by Brothers et al. [7], but we address it in this study.

An unbiased bycatch estimate would require separating birds bycaught at setting from those bycaught at hauling because of the differences in both loss rate and survival rate during these two processes. If the loss rate were applied to all the recorded bycatch, the estimate would over-shoot the underlying number, the other situation we want to avoid, since newly hooked seabirds at the line-hauling stage are not subject to the same bycatch loss process as set-captures and they can be counted reliably by the observer. Unfortunately, with most haul only observations, e.g., the POP in the western North Atlantic, it is impossible to assign without uncertainty where each recorded bycatch was originally hooked, because no direct observation on the origin of the bycatch currently exists. To solve this problem, we used the condition (or status as used in the POP) of the bycatch, which was available from the POP, to infer the origin of seabird bycatch based on the observations from Gilman et al. [17], which is the only published work to our knowledge that reports both the origin and the condition of the bycatch.

Little is known about the total fleet-level seabird bycatch in the western North Atlantic PLL fishery, partly because of the rarity of such events and subsequent low rate of data accumulation (i.e., only 158 birds, involving 83 out of 18,726 longline sets, were reported from 1992–2016 by the POP of the western North Atlantic United States PLL fishery [16]), but also because of potential bycatch loss. In the western North Atlantic, the POP managed by the Southeast Fisheries Science Center targets a coverage of 8% in recent years [16, 18]. Actual observer coverage, averaging 7.03% of total fleet effort, may not have been sufficient for such rare events, particularly considering only a small fraction appears at risk, i.e., 0.46% of all the observed longlines from the POP recorded seabird bycatch [19–22], and increasing coverage is expensive.

In this study, we build upon a traditional loss-free bycatch assessment model, incorporate two important components of the seabird bycatch process, i.e., the origin of capture (set or haul) and potential bycatch loss of a set-capture, and borrow needed information from other studies through a Bayesian approach. Our goal is to gauge the size of the bycatch loss problem and to provide a first estimate of loss-corrected fleet level total seabird bycatch to guide the conservation of seabirds in this region and shed light on the efficacy of the current haul-only observation protocol for monitoring seabird bycatch.

## Materials and methods

### Overview

**Fig 1:**
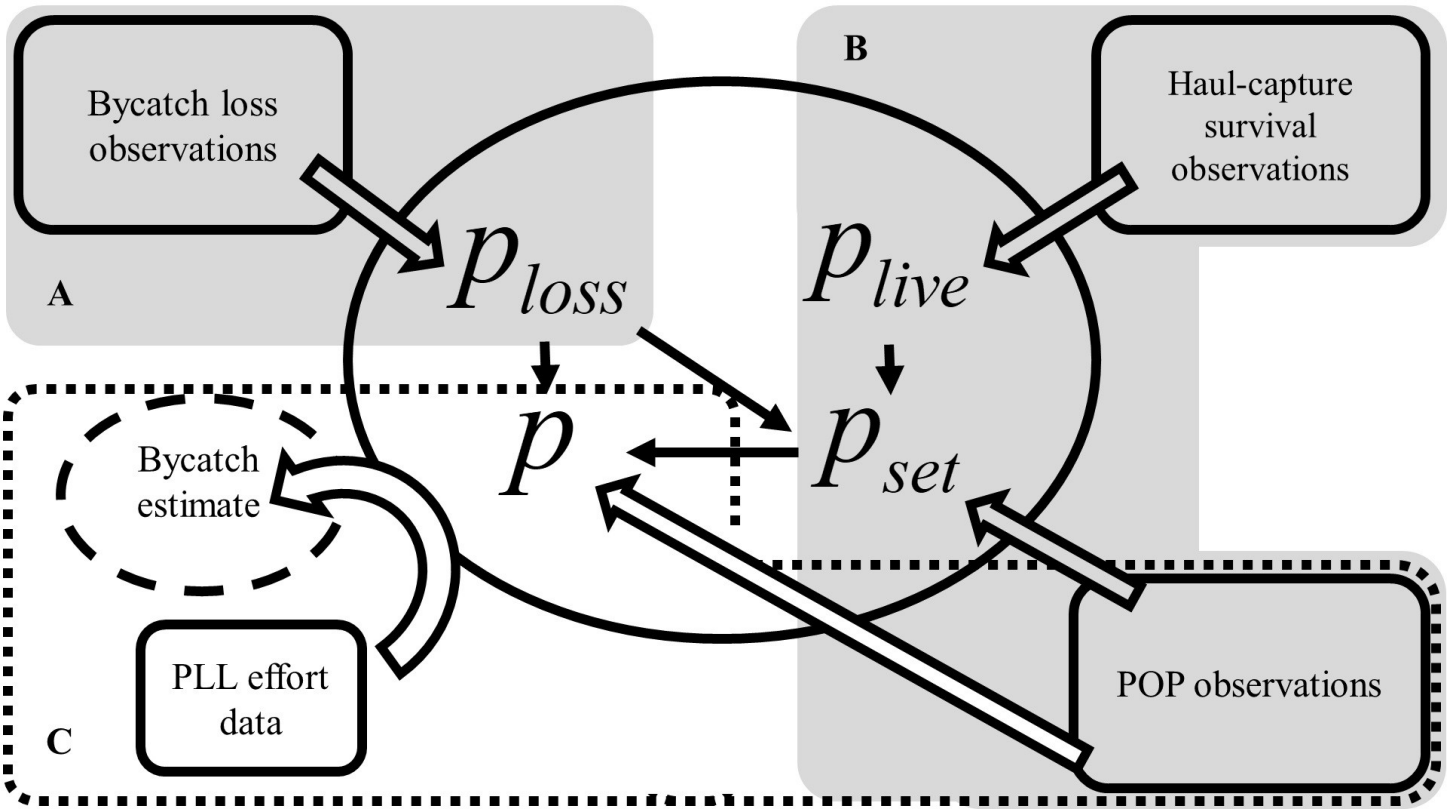
Organization of a seabird bycatch model and the associated data sources. The components of the integrated bycatch model include bycatch loss (A), the origin and the condition of the bycatch (B) and a traditional loss-free bycatch estimation and prediction model (C). Three data sources, Brothers et al. [7], Gilman et al. [17] and Atlantic POP (grey rectangles with solid borderlines), were employed to inform important parameters of the bycatch estimation model; U.S. Atlantic PLL logbook data were used to project the fleet level total bycatch. In particular, Brothers et al. [7] informed the estimation of bycatch loss rate (*P*_*loss*_) with details in Fig 2; Gilman et al. [17] and POP data together informed the estimation of the survival rate of haul-captures (*P*_*live*_) and the probability of set-captures (*P*_*set*_) with details in Fig 3.

In this study, the integrated seabird bycatch model has three major components: 1) bycatch loss of set-captures, 2) capture origin (set or haul) and 3) bycatch rate estimation and prediction model (Fig 1). A traditional loss-free bycatch estimation and prediction model would only contain component 3); components 1 and 2 are the major contributions of this study.

**Fig 2:**
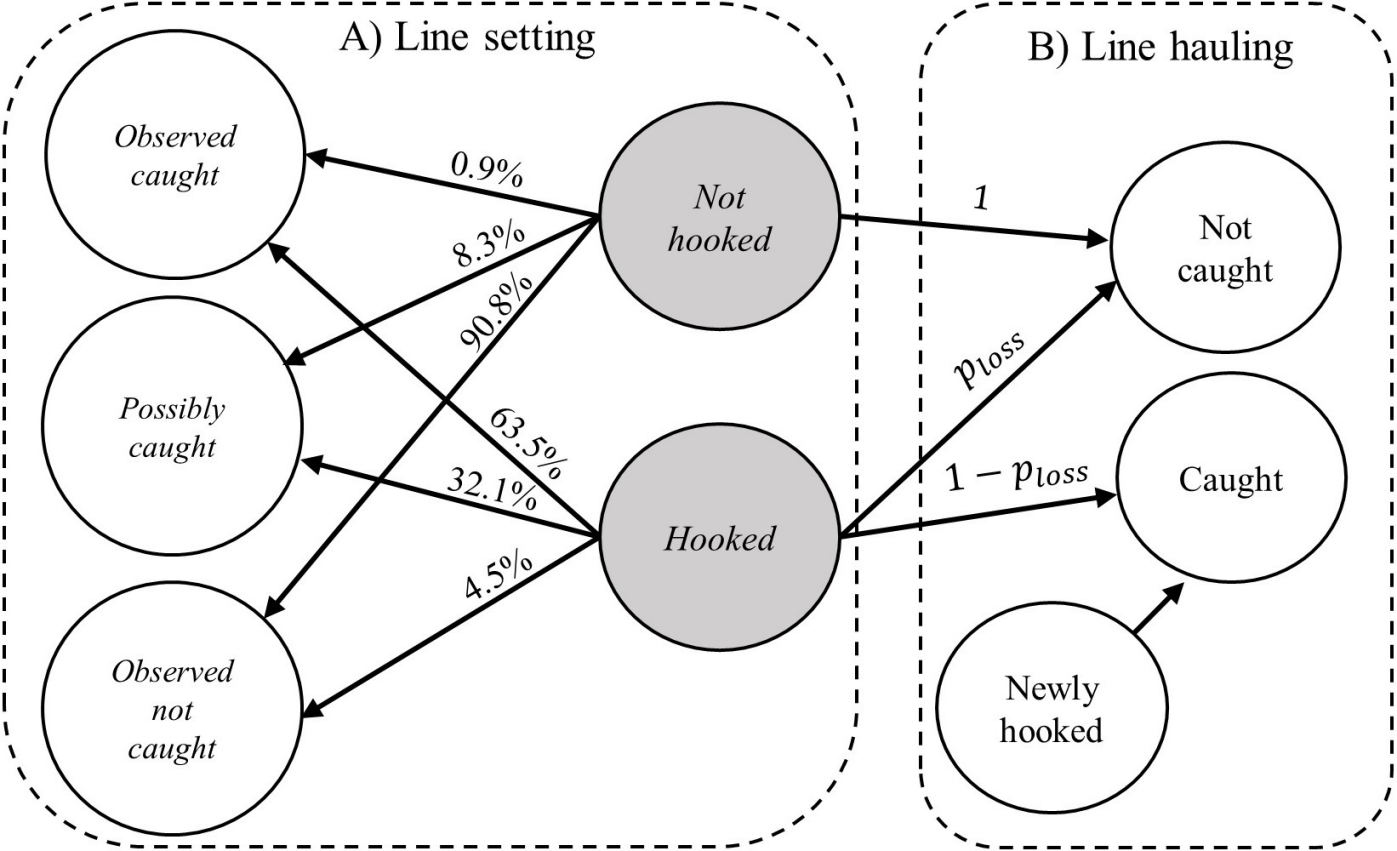
Classification of seabird interactions and subsequent carcass retrieval. At the line setting stage (A), bait-taking interactions of seabirds are classified into three broad categories, i.e., observed caught, possibly caught and observed not caught (clear circles on the left). The true state of the interaction, i.e., either hooked or not hooked (gray circles in the middle), is not directly observable to both the observers at the line setting and hauling stages. On the arrows running from a true state to a classified type are the estimated classification probabilities. For example, an interaction that does not lead to a hooking event (top middle circle) may be classified as observed not caught (lower left circle) with a 90.8% probability. Seabirds hooked during the line setting stage could drop off the hook with probability (*p*_*loss*_). Additional seabirds may get caught at the line hauling stage (newly hooked in B).

**Fig 3:**
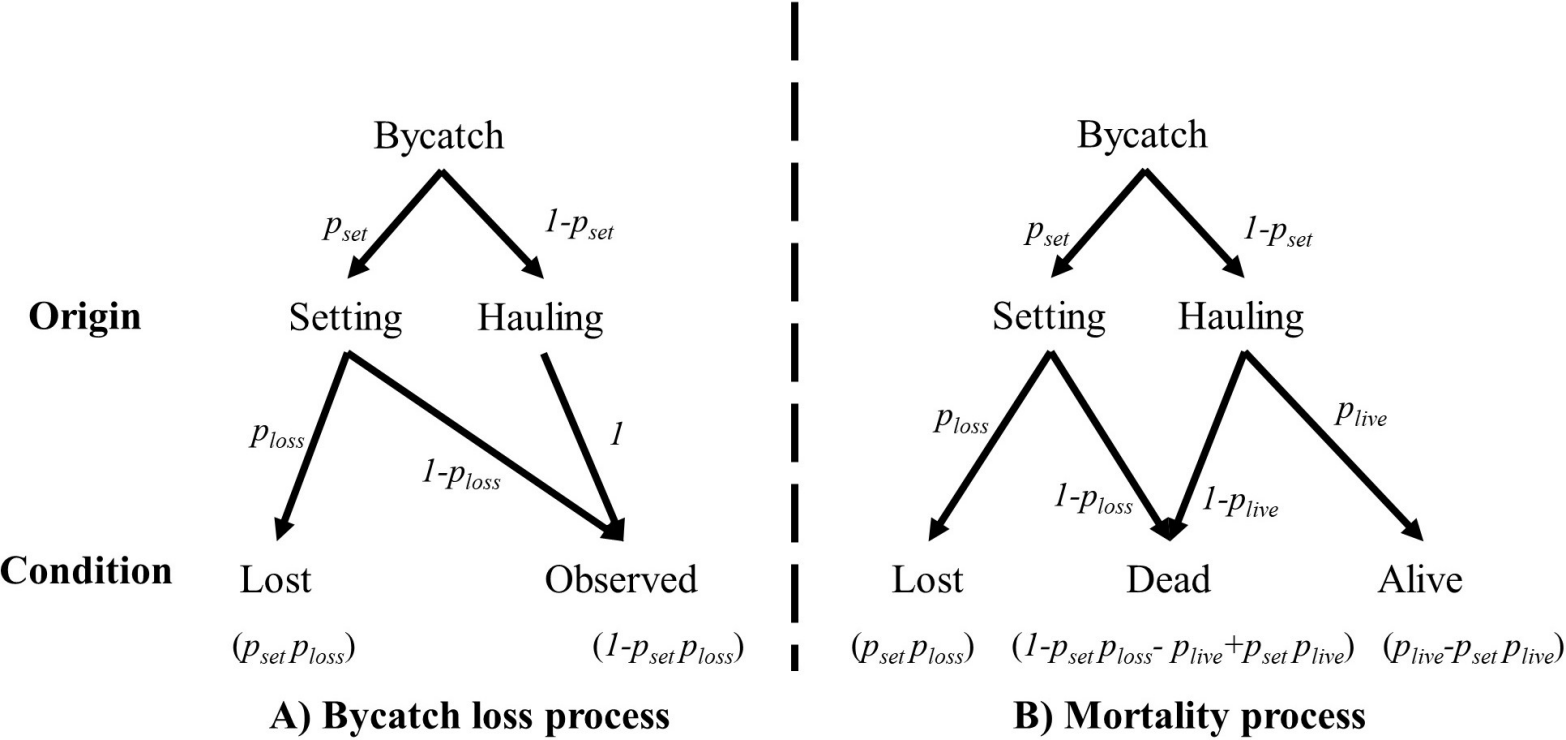
**Two-step processes for both the bycatch loss (A) and mortality (B).** The probability for each stage is in italics, and the probabilities of final outcomes can be found in parentheses under each outcome.

Four data sources were used in this study: 1) the POP observations, 2) seabird interaction types and hauled back carcass counts from Brothers et al. [7], 3) set- and haul-capture seabird mortality data from Gilman et al. [17], and 4) U.S. Atlantic PLL logbook data (Fig 1). Data from sources 1–3 were used to inform important parameters of the bycatch estimation model, and logbook data were used to project a loss-corrected total fleet level bycatch. The POP only observe a fraction of all the US longline operations in this region. The logbook data does not provide any seabird bycatch observations. Count data from Brothers et al. [7] informed the estimation of bycatch loss rate (*p*_*loss*_) (Figs 1A and 2); data from the POP and Gilman et al. [17] together informed the estimation of the survival rate of a haul capture (*p*_*live*_) and the probability of a set-capture (*p*_*set*_) (Figs 1B and 3).

### Pelagic Observer Program and U.S. Atlantic PLL fleet logbook data

The U.S. Atlantic PLL fishery primarily targets yellowfin tuna (*Thunnus albacares*), bigeye tuna (*Thunnus obesus*), other tuna species, swordfish (*Xiphias gladius*), sharks and dolphin fish (*Coryphaena hippurus*). A total of 18,913 longline sets were recorded in the POP from 1992 to 2016. Observers recorded not only bird bycatch, by species, but also the bycatch of many other incidental taxa, as well as target species. Environmental information, spatial coordinates, target species and set characteristics, such as number of hooks per set, were also recorded. The logbook data include environmental information, date, spatial coordinates, target species and set characteristics for each longline set.

Both the Atlantic POP and PLL logbook data from 1992 to 2016 were provided by the Fisheries Statistics Division of the Southeast Fisheries Science Center of the National Marine Fisheries Service, Miami, Florida. Records with obviously erroneous or missing necessary information, such as geographic coordinates or number of hooks, were excluded from the analysis. A total of 18,726 longline sets with 83 sets of positive bycatches from the observer data were used for model fitting, and a total of 266,324 longline sets from the logbook data were used to estimate the total bycatch.

### Bycatch loss of set-captures

Counts of line setting and hauling observations were extracted from Brothers et al. [7] (Table 1). Bait-taking interactions can be classified into three broad categories based on the expected outcome of the interaction and the observer’s confidence about the outcome: 1) the observer is certain that the seabird is caught (observed caught in Table 1); 2) the seabird may get caught but the observer is uncertain of the outcome (possibly caught in Table 1); 3) the observer is certain that the seabird is not caught (observed not caught in Table 1). Note that Brothers [3] originally classified the interactions into five categories, but only presented the aggregated counts from three broad categories [7]. Spatial variation of the observation was not modeled, because the published observation counts were aggregated across all regions. Obtaining greater detail from existing observation data would have yielded more information on the variability of the bycatch loss rate. Instead, we used the aggregate loss rate as a reference and adopted a scenario analysis approach, as described in the Bayesian method section, as a first estimate of the impact of bycatch loss on the total fleet bycatch in the western North Atlantic.

**Table 1:** Counts of bait-taking interaction categories and the associated counts of retrieved carcasses from Brothers et al. [7].

Bait-taking interaction types	*Observed caught*	*Possibly caught*	*Observed not caught*
# of interactions (# carcasses retrieved) *	176 (85)	553 (42)	5,367 (5)

Bait-taking interactions at the line setting stage were classified into 1) observed caught, when the observer is certain that the seabird is caught, 2) possibly caught, when seabird may be caught but the observer is uncertain of the outcome, and 3) observed not caught, when the observer is certain that the seabird is not caught.

*: **# of interactions** is the recorded number of interaction between seabirds and longline gear; **# carcasses retrieved** in parentheses is the number of hauled-back seabird bycatch resulting from those recorded interactions.

Classification of the bait-taking interactions of seabirds with longline gear is subject to observation error, i.e., misclassification. It is of interest to know the amount of uncertainty in the classification process, e.g., the probability of misclassifying an interaction that resulted in a hooking event into the possibly caught category. In the following, we derive the observation model based on the rules of probability, and the text may look convoluted because two sets of observations are involved, 1) interaction type classification during line setting and 2) seabird carcass retrieval during line hauling (Fig 2). The purpose of using an observation model is to separate the classification error from the variability of the underlying bycatch loss rate (*p*_*loss*_), which was later linked to other components of the model to obtain the loss-corrected bycatch estimate (Fig 1).

The classification of interactions at line setting (clear circles in Fig 2A) and seabird carcass retrieval at line hauling (clear circles in Fig 2B) are linked through the unobserved hooking events (shaded circles in Fig 2A). Since the true hooking events at line setting are not directly observable, observations may either originate from a “hooked” or “not hooked” event, and the probability of the observation involves the sum from these two cases. The probability of classifying an interaction into an *observed caught* category is

$$\begin{array}{l} \text{Prob}(Hooked)\times \text{Prob}(Hooked\to Observed\;caught)+\\ \text{Prob}(Not\;hooked)\times \text{Prob}(Not\;hooked\to Observed\;caught), \end{array}$$

where Prob(*Hooked*) is the unobservable event denoting the probability of a recorded interaction leading to a hooking event, Prob(Hooked→Observedcaught) denotes the probability of classifying an interaction that resulted in a hooking event into the *observed caught* category. The probability of classifying an interaction associated with a hauled-back bycatch into the *possibly caught* category is

$$Prob(Hooked)\times Prob(Hooked\to Possibly\;caught)\times (1-p_{loss}).$$

The probability of classifying an interaction associated with a hauled-back bycatch into the *observed not caught* category is then

$$Prob(Hooked)\times Prob(Hooked\to Observed\;not\;caught)\times (1-p_{loss}).$$

### Capture origin

Due to potential bycatch loss between line setting and line hauling, seabird bycatch is only partially observable at the line hauling stage, so the observed bycatch was modeled as an integrated two-step process in relation to the underlying probability of the bycatch (Fig 3A). A seabird can be caught either at the line-setting stage (we denote this probability as *P*_*set*_) or at the line-hauling stage, and then set-captures can drop off the hook with a probability of *P*_*loss*_ and become unobservable. On the other hand, haul-captures were assumed be perfectly observed (See discussion for other scenarios). Therefore, each underlying bycatch can be observed with a probability of $1-p_{set}\times p_{loss}$.

To obtain the probability of observing a positive bycatch, we need to add up the probabilities of catching *i* seabirds and losing *i-k* of them, where *K*≤*i*. Assuming that each bycatch event was independent of any other bycatch caught on the same longline, the probability of observing a positive bycatch *K*≥*i* is binomially distributed with

$$Prob(x=k)=\sum_{i\geq k}C_{k}^{i}\times Prob(n=i)\times {\left(1-p_{set}p_{loss}\right)}^{k}\times {\left(p_{set}p_{loss}\right)}^{i-k},$$

where *n* is the underlying number of seabird(s) caught including those lost and unobserved seabirds, *x* is the observed bycatch, and $C_{k}^{i}$ is a binomial coefficient denoting the number of ways to choose *k* item(s) from a set of *i* item(s). Likewise, the probability of no seabird bycatch during line setting, i.e., Prob(x = 0), is thus the sum of probability of catching no seabirds 1-*p* and the probabilities of catching and losing *i* seabirds, i.e.,

$$Prob(x=0)=1-p+\sum_{i\geq 1}Prob(n=i)\times {\left(p_{set}p_{loss}\right)}^{i}.$$

Some birds bycaught after the hauling are still alive, and field observations suggested that most birds caught during the setting stage are dead due to extended soaking time with some exceptions as noted in Brothers [23]. The average soak duration in the U.S. Atlantic PLL fleet is 8.35 hours according to the POP data. In this study, we assume 100% mortality for set-captures upon retrieval. The condition of the bycatch was modeled with an underlying two-step process (Fig 3B). Given that a bycatch is observed, the probability that it is dead upon retrieval is $(1-p_{set})\;p_{live}$, and the probability that it is alive is $1-p_{set}p_{loss}-p_{live}+p_{set}p_{live}$. A region-specific estimate of haul-capture is not available, and we used the observations from Gilman et al. [17], the only reference known to us that published both origin and condition of the bycatch. It is assumed that haul-captures in the Atlantic have a similar mortality rate as those from Gilman et al. [17]. A total of 16 seabirds were observed caught during line hauling, and all were alive upon retrieval [17]. Because of the small sample size (16), instead of assuming all seabirds caught during line hauling are alive upon retrieval we added small sample uncertainty to the model by giving a noninformative prior of *Beta*(1,1) to the probability that a seabird caught during line hauling was alive upon retrieval. Then we integrated the mean survival rate estimate into the bycatch model to infer the probability of set-captures.

There is incidental evidence in the Hawaii pelagic longline fishery that some robust bird species bycaught during the setting stage may survive the soak period and be hauled aboard alive [23]. To accommodate this scenario, only modifications to the mortality process are required (See **S1 File** for details). However, in this study, we did not consider set-capture survival, because the publicly available bycatch mortality data from Gilman et al. [17] indicates 100% set-capture mortality (96 dead out of 96 coming up from the soak).

### Bycatch rate estimation

Most records (over 99%) of the Atlantic POP data set did not record a seabird bycatch, and hurdle models were commonly used to account for these excess zero records [24–26]. In this study, we also use hurdle models, and in the following, we describe how the binary process and the count process were modeled.

The binary process models the probability of catching one or more seabirds in one longline operation. The occurrence or absence of a seabird bycatch (Y) was modeled to follow a Bernoulli random process

Y~Bernoulli(p),

where *p* is the probability of a seabird bycatch event, and *p* was modeled with respect to factors related to fishing operations, spatial and seasonal factors, and inter-annual changes,

$$logit(p)=c_{b}+X_{fishery}\times \theta_{fishery}+X_{season}\times \theta_{season}+X_{spatial}\times \theta_{spatial}+X_{year}\times \theta_{year},$$

where $c_{b}$is an intercept, $X_{fishery}$ is the categorical factors of the target species of the longliner, $X_{season}$ is the categorical seasonal factors, $X_{spatial}$ is the numerical covariates of the coordinates of the fishing location (rescaled to have a zero mean and unit variance), $X_{year}$ is the categorical factors representing the year of the fishing operation, and *θ*s are the respective coefficients to be estimated. The set of covariates for the binary model was based on the hypothesized significance of those variables in predicting seabird bycatch and the availability of such records in the data [24, 27]. Coordinates are used to account for the large scale spatial pattern of seabird bycatch in this region; the season variable was used to account for changes in seabird abundance in this region due to migration; target species type was used to account for potential bycatch differences related to fishing tactics; year effect was modeled as a random effect because of the better performance of random year effect models based on a simulation study [24, 27–29].

The counting process models the positive seabird bycatch events. K-aggregated Conway-Maxwell-Poisson (CMP) distributions were used due to its superior performance in reducing bias in predicting positive catch data over traditional models such as log-normal, Poisson and negative binomial distributions [30]. The CMP distribution is a generalization of the Poisson distribution, and, with one additional shape parameter, it can model both over-dispersion and under-dispersion [31–33]. Here, the Lord et al. [32] formulation of the CMP was used

$$f_{CMP}(y=n)=\frac{1}{S(\mu ,\nu )}{\left(\frac{\mu^{n}}{n!}\right)}^{\nu },$$

$$S(\mu ,\nu )=\sum_{i=0}^{\infty }{\left(\frac{\mu^{i}}{i!}\right)}^{\nu },$$

where S(μ,ν) is a normalizing constant, ν≥0 is the shape parameter, *n* is the observed count, and μ>0 is a centering parameter of the CMP distribution and was assumed that log(*μ*) has a linear relationship with the covariates. Due to the small sample size of positive bycatch records, only one covariate, the number of hooks, was used in the counting process sub-model. The number of hooks deployed is a frequently used measure of PLL fishing effort in bycatch studies [11, 17, 34]. The *k*-aggregation method has been shown to improve model fit to count data of rare events, and this method involves mapping the probability of singleton counts (n = 1) to the sum of the initial k+1 probabilities of a baseline distribution over the positive range, and the probability of two or more counts (n≥2) is mapped to the probability of the count of *k+n* of the baseline distribution [30]. In this way, parameter *k* was used as an additional shape parameter of the distribution to adapt to the observed data pattern, and the model with *k* = 0 corresponds to the original baseline distribution. The *k*-aggregated CMP distributions with different *k* values were named CMP[*k*].

### Bayesian method

A Bayesian approach was used for parameter estimation. We used wide normal priors with mean zero on rescaled covariates, and uniform priors for distribution-specific shape parameters. To simulate MCMC (Markov Chain Monte Carlo) samples from the posterior distribution, we used JAGS 4.0 [35] in the statistical program R 3.2.5 [36]. Model performance was measured based on deviance information criterion [37]

$$DIC=\bar{D}+pD,$$

where deviance *D* is twice the negative log-likelihood, $\bar{D}$ is the posterior mean of the deviance, and pD is an estimate of the effective number of parameters in the model based on the algorithm proposed by Plummer [38]. A model with lower DIC outperforms a model with higher DIC [39].

Four bycatch loss scenarios were simulated, i.e., low, medium, high and expected bycatch loss. The first three scenarios correspond, respectively, to the estimates of bycatch loss rate (*p*_*loss*_) at the 2.5th, 50th and 97.5th percentiles of the posterior estimates of the bycatch loss rate based on observations from Brothers et al. [7]. These estimates were used in the bycatch estimation model to estimate the other parameters and predict total bycatch from those scenarios. These scenarios tell us the magnitude of the bycatch loss problem without considering its uncertainty. In the fourth scenario, the expected bycatch loss scenario, we estimated the overall bycatch and uncertainty using a Monte Carlo method to integrate out bycatch loss rate (*P*_*loss*_) from the bycatch estimation model. We drew a random sample of 200 bycatch loss rates from the posterior sample of *P*_*loss*_, independently fitted bycatch estimation models with *P*_*loss*_ set to those respective values, and then pooled the posterior estimates from these 200 models to get the final estimates. Thus, the expected bycatch loss scenario incorporates both the possibility of a bycatch loss and its estimation uncertainty given the current data situation.

## Results

The misclassification rate of the seabird interactions with fishery by Brothers et al. [7] is relatively low (Table 2). On average, 95.6% of set-captures can be correctly classified as either *observed caught* (63.5%) or *possibly caught* (32.1%), and 90.8% of non-capture interactions can be correctly classified as *observed not caught*. The number of observations in the not caught category was relatively high, and a small percentage of false positives (8.3%) can obscure the information on bycatch loss rate.

**Table 2:** Estimated posterior mean and 95% credible interval of the classification probabilities (in %) of seabird interactions with fisheries.

Classification	Underlying state	
	Hooked	Not hooked
*Observed caught*	63.5 (55.4, 71.8)	0.9 (0.0, 1.6)
*Possibly caught*	32.1 (24.4, 40.2)	8.3 (7.5, 9.1)
*Observed not caught*	4.5 (1.4, 8.0)	90.8 (89.5, 92.2)

**Fig 4:**
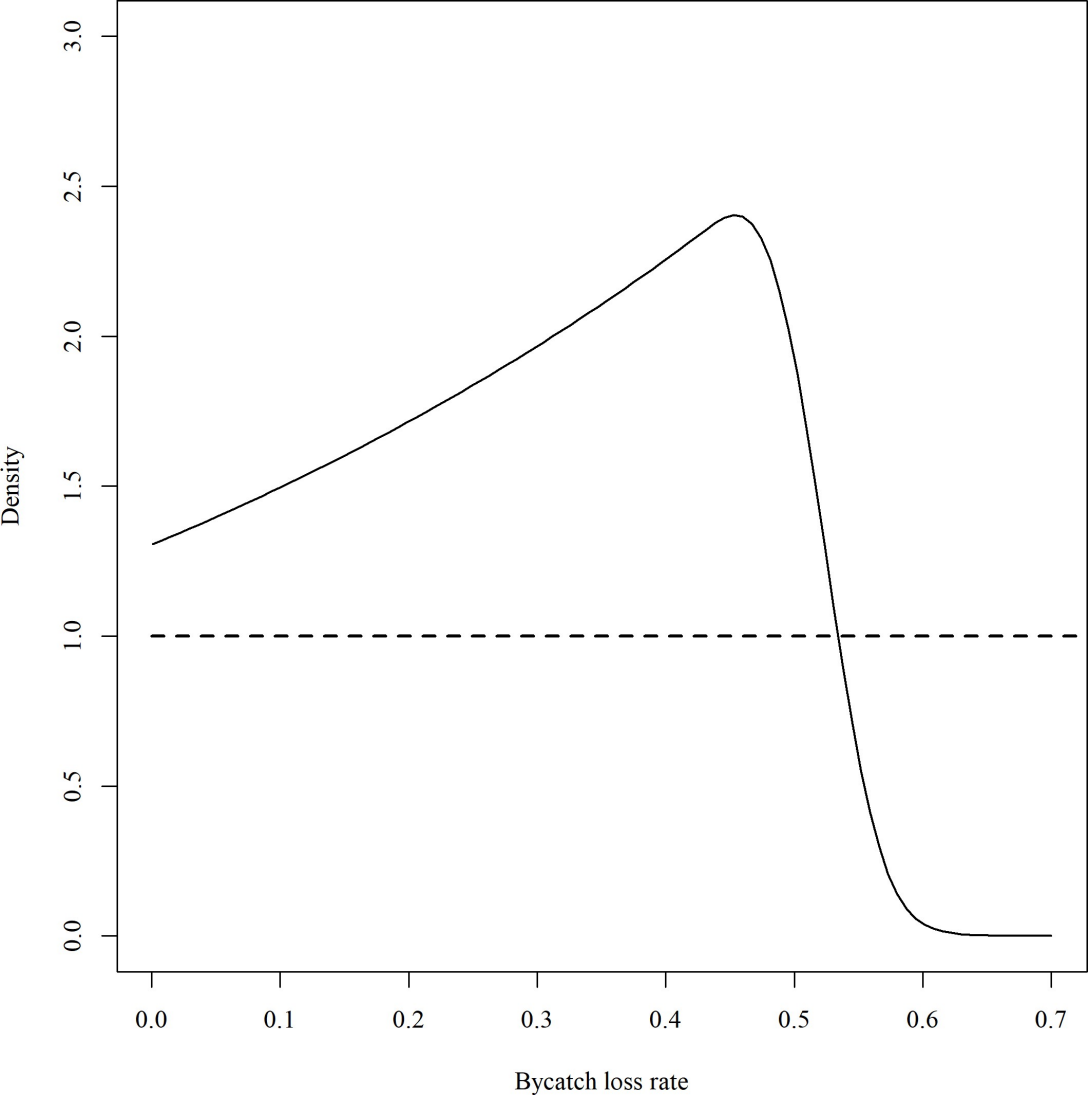
Prior (dashed line) and posterior (solid line) distributions of the bycatch loss rate (*p*_*loss*_). The prior is a uniform distribution from 0 to 1. Count data extracted from Brothers et al. [7] were used to estimate *p*_*loss*_.

Considering misclassification, bycatch loss rate of set-captures (*p*_*loss*_) was estimated to be on average 29.8 percent with a 95% credible interval of (0.24, 51.88) percent (Fig 4). This estimate is smaller than the raw estimate in Brothers et al. [7], which did not consider uncertainty in the observation. The survival rate of haul-captures (*p*_*live*_) was estimated at on average 94.4 percent with a 95% credible interval of (80.5, 99.9) percent.

**Fig 5:**
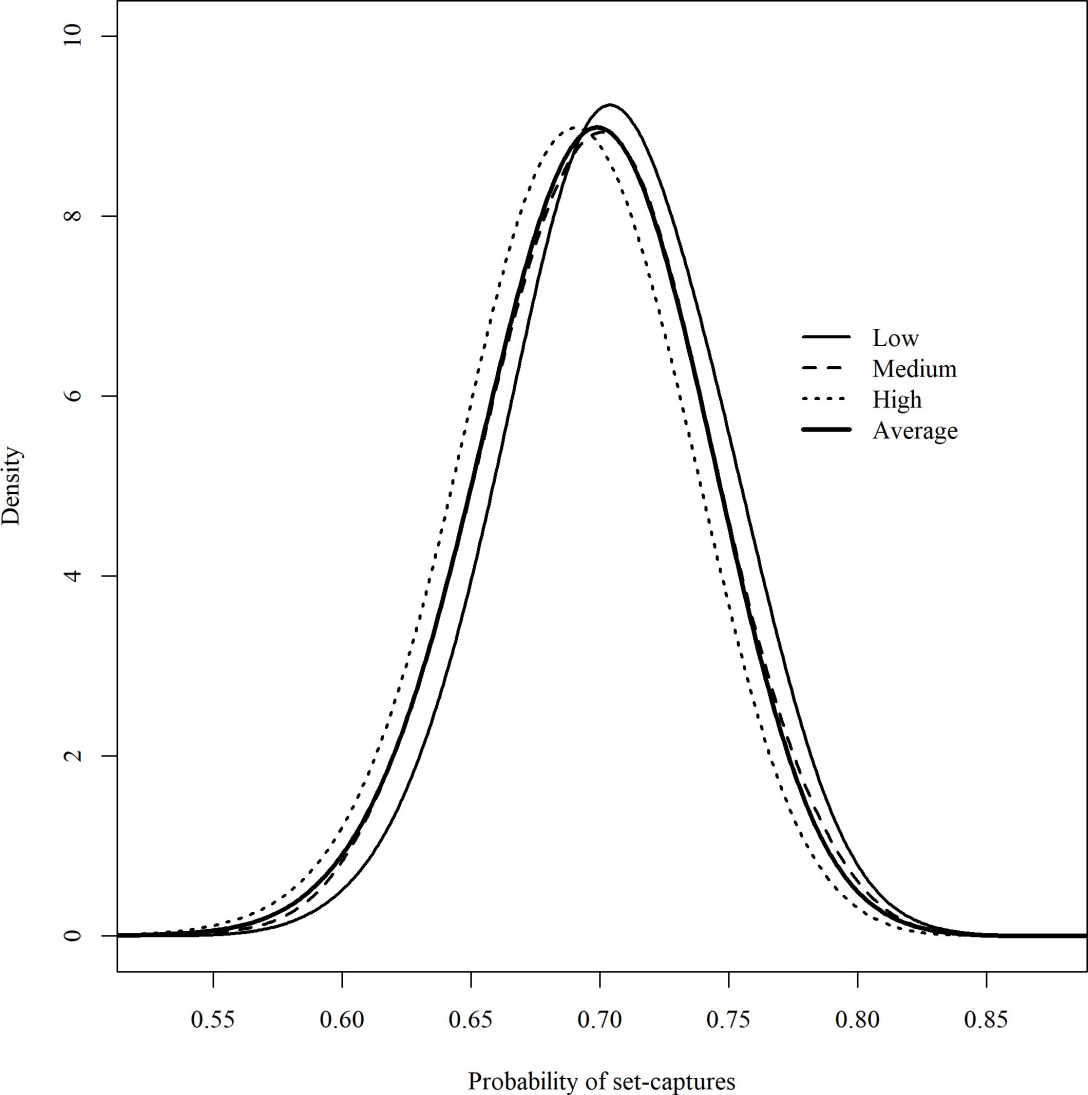
Probability of set-capture (*p*_*set*_) in the U.S. Atlantic PLL fleet for different bycatch loss scenarios. The low, medium and high bycatch loss scenarios use, respectively, point estimates of bycatch loss rate (*p*_*loss*_) at the 2.5th, 50th and 97.5th percentiles of the posterior distribution of the bycatch loss rate based on observations from Brothers et al. [7], and the expected bycatch loss scenario uses the entire posterior distribution of *p*_*loss*_ in the calculation.

The bycatch model with a modified CMP distribution was selected, based on DIC, for the low, medium, and high bycatch loss scenarios (Table 3). Compared to the original CMP distribution, use of the modified CMP distribution substantially improved model fit with a reduction of 14.4 to 15.9 in DIC. Based on the selected models, most seabird bycatch in the Atlantic, including both observed and lost bycatch, occurred at the line-setting stage. Less than 1% of the posterior samples of the probability of set-captures (*p*_*set*_) were smaller than 50% for all bycatch loss scenarios (Fig 5). The estimated probability of set-captures was similar among all bycatch loss scenarios, e.g., on average, *p*_*set*_ = 70.5% for the low bycatch loss scenario, 69.8% for the medium bycatch loss scenario, 68.9% for the high bycatch loss scenario, and 69.6% for the expected bycatch loss scenario.

**Table 3:** Model selection summary for the low, medium and high bycatch loss scenarios.

Bycatch loss scenario	Count distribution	ΔDIC
Low	CMP	14.4
	CMP.1	0.0
	CMP.2	1.4
	CMP.3	1.4
Medium	CMP	15.9
	CMP.1	1.9
	CMP.2	0.0
	CMP.3	1.9
High	CMP	14.8
	CMP.1	5.0
	CMP.2	0.9
	CMP.3	0.0
	CMP.4	2.8

Candidate models under each bycatch loss scenario are identical except for the count distributions used for the positive catch. The count distribution CMP denotes the original Conway-Maxwell-Poisson distribution, and CMP[*k*], where k = 1,2…, denotes a modified Conway-Maxwell-Poisson distribution with the additional shape parameter *k*.

**Fig 6:**
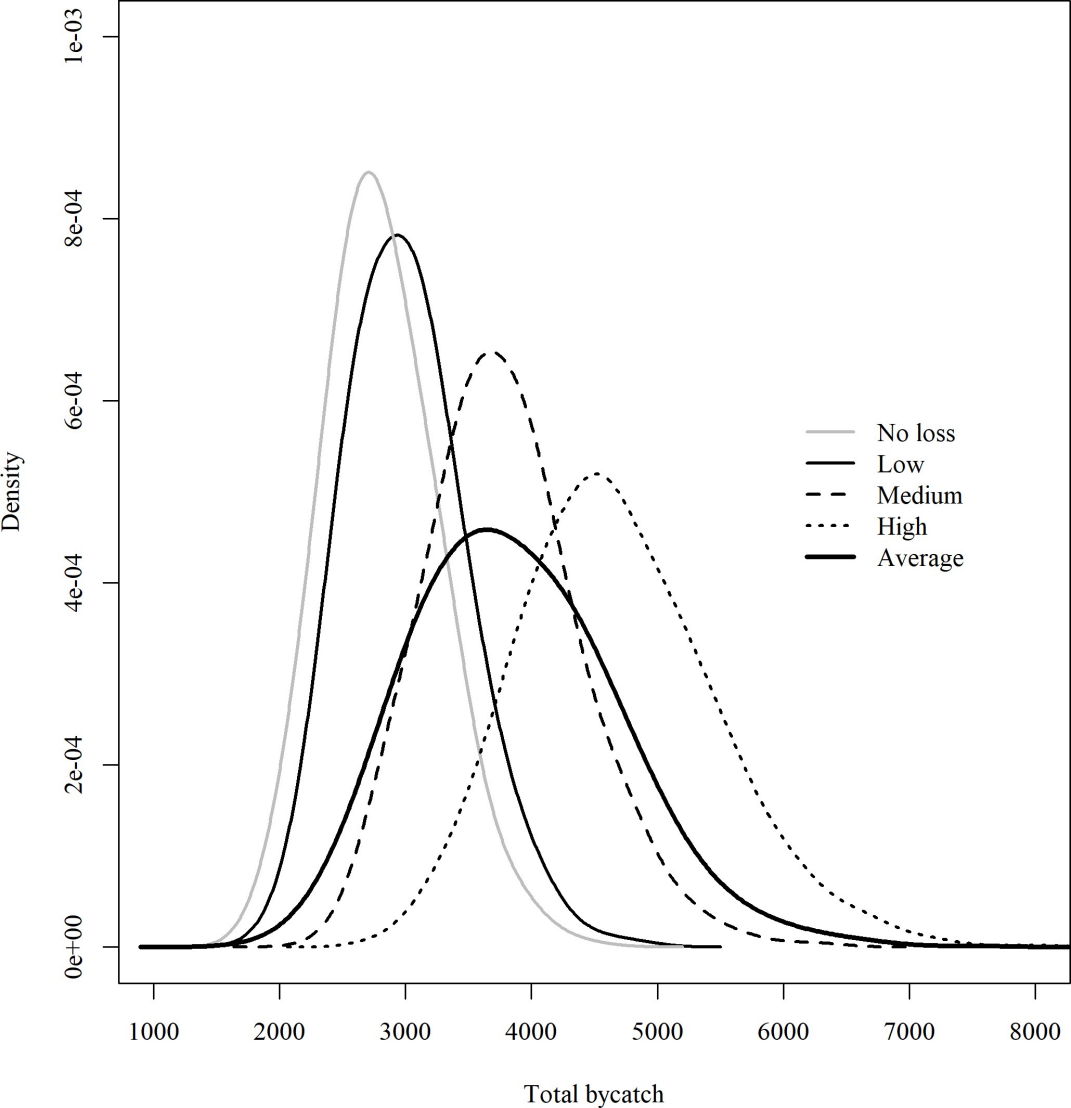
Estimated total seabird bycatch from 1992 to 2016 by the U.S. Atlantic PLL fleet. Estimates of total seabird bycatch were generated for four bycatch loss scenarios and a loss-free model.

The selected bycatch model for each scenario was used with logbook data to project total seabird bycatch at the fleet level. For the low bycatch loss scenario, during the entire study period (1992–2016), the total bycatch is projected to be 3,005 seabirds on average with a 95% credible interval of 2,194 and 4,024 seabirds; for the medium bycatch loss scenario, the total number of seabirds caught by the U.S. fleet is estimated at 3,787 seabirds on average with a 95% credible interval of 2,752 and 5,121 seabirds; for the high bycatch loss scenario, the total bycatch is projected to be 4,686 seabirds with a 95% credible interval of 3,312 and 6,437 seabirds; and for the expected bycatch loss scenario, the total bycatch is projected to be 3,801 seabirds with a 95% credible interval of 2,511 and 5,664 seabirds (Fig 6). The uncertainty in the projected estimate, measured by both the standard deviation (std) and the coefficient of variation (CV) of the projected total bycatch, increased with the assumed bycatch loss rate (Table 4). The shape of the distribution of posterior estimates of total bycatch was relatively narrow for the low bycatch loss scenario and gradually widened moving to the high bycatch loss scenario and the expected bycatch loss scenario, showing the increase in uncertainty measured by std and CV. With a higher loss rate, a larger proportion of the total bycatch becomes unobservable, thus further inflating the uncertainty in the estimated parameters and, subsequently, the projected total bycatch. The expected bycatch loss scenario has the largest CV among all the scenarios because this scenario incorporates both the uncertainty induced by the bycatch loss process and the uncertainty in the estimation of *P*_*loss*_.

**Table 4:** Mean, standard deviation (std.) and coefficient of variation (CV) of projected fleet level total (1992–2016) seabird bycatch from different bycatch loss scenarios in the Western North Atlantic.

Bycatch loss scenarios	Projected total bycatch		
	Mean	Std.	CV
No loss	2,813	438	0.1558
Low loss	3,005	471	0.1566
Medium loss	3,787	609	0.1607
High loss	4,686	784	0.1673
Expected loss	3,869	807	0.2088

**Fig 7:**
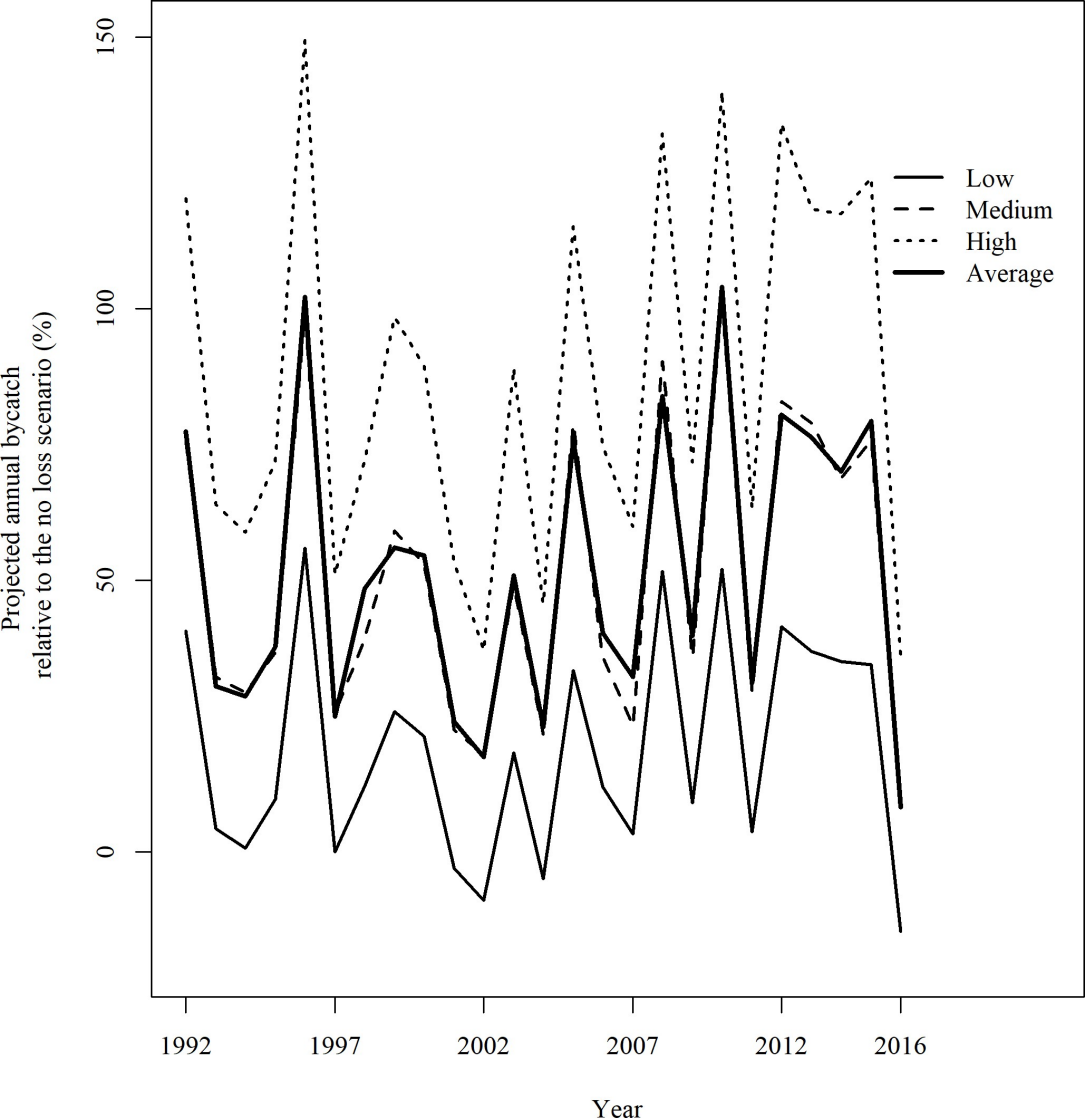
Relative differences in annual bycatch between different bycatch loss scenarios and the loss-free model.

Compared to the loss-free bycatch model, the models with bycatch loss produced disproportionally more bycatch than the corresponding loss rate used. For the low-bycatch-loss scenario, with a loss rate of 0.24%, the projected total bycatch was on average 9.5% higher than the reference model; for the medium loss scenario, with a loss rate of 29.8%, the projected total bycatch was on average 37.9% higher; and for the high loss scenario, with a loss rate of 51.9%, the projected total bycatch was on average 70.6% higher (Table 3). The percentage of relative mismatch from the loss-free model varied across years, and in some years the mismatch was substantially higher than the average (Fig 7). For example, in 1996 the projected annual bycatch from the low, medium and high bycatch loss scenarios was 55.9%, 98.9% and 149.3% higher, respectively, than bycatch estimated by the loss-free model.

## Discussion and conclusion

Seabird bycatch databases for the U.S. PLL fishery in the western North Atlantic rely on observations of seabirds brought to the deck when the line is hauled back to the vessel and ignore seabird captures that fall off the hook before reaching the vessel. According to this study, the bycatch loss is substantial. The current observer data collection protocol and conventional loss-free bycatch estimation methods substantially underestimate both total fleet seabird bycatch and estimation uncertainty.

The purpose of this study was to gauge the size of the bycatch loss problem and provide a first estimate of the loss-corrected fleet total bycatch. With the region-specific input, the methodology presented here could be used to obtain a more accurate estimate of total bycatch than is being obtained today. Our estimated bycatch loss rate was based on observation data in other distant oceans, i.e., Indian Ocean, Southern Ocean, Coral Sea and Central Pacific Ocean, as no such observations have been conducted in the Atlantic Ocean [7–9]. Seabird bycatch loss rate during the soak period may be influenced by several factors, such as bird behavior, predator community and fishing tactics including the type of gear used; therefore, the loss rate may differ among fleets [7]. To partially compensate for this data non-specificity, we explored four substantially different bycatch loss scenarios, using a loss rate as low as 0.24% and as high as 51.88%. It is unclear which scenario best suits the situation in the western North Atlantic, and the loss rate in this region may well exceed the maximum loss rate explored here, but the point is that all four bycatch loss scenarios suggest significantly more bycatch and more estimation uncertainty than is estimated without taking loss rate into account. A specific line setting and hauling observation component as in Brothers et al. [7] would be extremely helpful in quantifying a region-specific seabird bycatch loss rate in the Atlantic. According to this study, most seabird bycatch (approximately 70% on average) was inferred to have been caught at the line setting stage. Before they can be observed after hauling, these bycatches are subject to loss [8, 40–42]. In a Hawaii deep-set pelagic longline tuna fishery, the seabird carcasses coming from the soak dominate the seabird bycatch [17]. This further highlights the urgency of adopting a loss-corrected modeling approach for bycatch estimation and modifying the present haul-only observation protocol for seabird bycatch monitoring purposes.

This study provides a first estimate of the impact of seabird bycatch loss on estimates of full-fleet bycatch and reveals potential risks associated with the current observation protocol and the loss-free assumption in bycatch projection. The downside of this assessment is that the bycatch loss rate we used is not specific to the western North Atlantic Ocean region, as no at-sea observations on bycatch loss have been conducted in the Atlantic Ocean. The new modeling framework presented here builds upon a traditional loss-free bycatch model and loss-free haul-back data but incorporates other crucial components of the seabird bycatch process, i.e., origin (line-set or line-haul), loss of set-captures, and condition (live or dead) of capture. This framework can readily be adapted to other PLL fisheries to provide a first estimate of the fleet level loss-corrected seabird bycatch using traditional observer data from a given fishery and data acquired elsewhere from more comprehensive observer coverage.

We urge other researchers to adopt a loss-corrected modeling approach for bycatch estimation and consider revising the current observer protocol to quantify potential seabird bycatch loss, as also urged by Brothers et al. [7] and Gilman et al. [17]. There are considerable issues to consider in attempting the set and haul observations in this region, and it may only be practical here to use the bycatch loss observations from other oceans to build on haul-only bycatch through a modeling approach as we have done. Acquiring region-specific observations for other pelagic longline fisheries may also be problematic, and the method we have demonstrated here, coupled with the available data from Brothers et al. [7] and Gilman et al. [17], may provide a viable next best approach for these other regions.

The field of seabird bycatch analysis is hindered by the combination of an extremely low rate of occurrence of bycatch and the lack of comprehensive at-sea observations, especially in multi-taxa monitoring programs, such as the POP. In our model, the origin of the observed bycatch was inferred based on seabird condition and haul-capture mortality. State can be directly observed, e.g., a completely soaked seabird carcass being hauled into the vessel can only have been caught during line setting [17]. This additional observation is already captured on an existing form (in use since 2005) that is filled out onboard by the attendant observer for every bird caught. However, information from this form presently does not enter the POP database that is used as the basis for estimating total fleet bycatch; furthermore, the necessary information box might not always be filled in. Training observers for collecting the required data carefully would reduce this type of problem in the data. A review of the photos that observers are supposed to take on each bird caught could substantiate or substitute for missing information on the form. The bird form wording should be revised to improve clarity with respect to whether a thoroughly soaked dead bird has been brought onboard, and training sessions with observers should be thorough in explaining this part of the form and why it is needed. Then a separate database of data from the bird form should be provided to future analysts to be incorporated into special analyses of data from 2005 forward.

We recommend an integrated modeling approach to account for bycatch loss. This may be the first time that seabird bycatch loss rate has been rigorously integrated into a bycatch estimation model. Accounting for sample size and false positives, the loss rate of set-captures was on average 29.8%, smaller than the raw value (52%) reported by Brothers et al. [7], and this loss rate translated into on average 37.9% more projected annual bycatch by the U.S. Atlantic PLL fleet than estimated by a model assuming no bycatch loss. Even a loss rate as low as 0.24% as in the low bycatch loss scenario translated into on average 9.5% higher total bycatch than the no bycatch loss projection. The relative difference between projected total bycatch and the no loss scenario also varied between years (Fig 7). The within-fishery difference in the relationship between seabird bycatch and fishing effort due to spatially and temporally varying intrinsic and external factors, as well as the non-linearity between loss rate and projected bycatch loss, necessitates a model-based approach to project the total bycatch from the observed records to the fleet level, rather than just dividing the estimate from a conventional loss-free model by the bycatch retention rate.

## Disclaimer

The scientific results and conclusions, as well as any views or opinions expressed herein, are those of the authors and do not necessarily reflect those of NOAA or the U.S. Department of Commerce.

## Supporting information

S1 File S1_File. (DOCX)
