# Supplementary material for: How much do we know about seabird bycatch in pelagic longline fisheries? A simulation study on the potential bias caused by the usually unobserved portion of seabird bycatch
Source: PLoS One. 2019 Aug 5;14(8):e0220797. doi: 10.1371/journal.pone.0220797 (PMC6681973; doi:10.1371/journal.pone.0220797)
Supplement: S1 File — (DOCX) [file pone.0220797.s001.docx]

## S1 File: Additional methods

## A general mortality process

The mortality process developed in the main text assumes 100% mortality for the set-captures, but there exists anecdotal evidence of some set-captures may survive the soak and be hauled aboard alive, for example, large robust species caught on lightly weighted shallow set lines. To accommodate such case, we need to generalize the mortality process presented in the main text. With a non-zero survival rate for set-captures (*p_s.live_*), both set- and haul-captures contribute to the bycatch recorded as alive at the haul (**S1 Figure**). When *p_s.live_* is set to zero, the mortality process is equivalent to the one presented in the main text. Other parts of the model do not require modification because, in this model component, only set-capture probability (*p_set_*) is interfacing with other components of the model (Fig 1). This general mortality process would be useful for those species with a documented positive set-capture survival rate, especially for bycatch assessments at the species level. In the main text, publicly available bycatch mortality observations indicate 100% set-capture mortality [1], and therefore a simplified mortality process was used in the analysis.


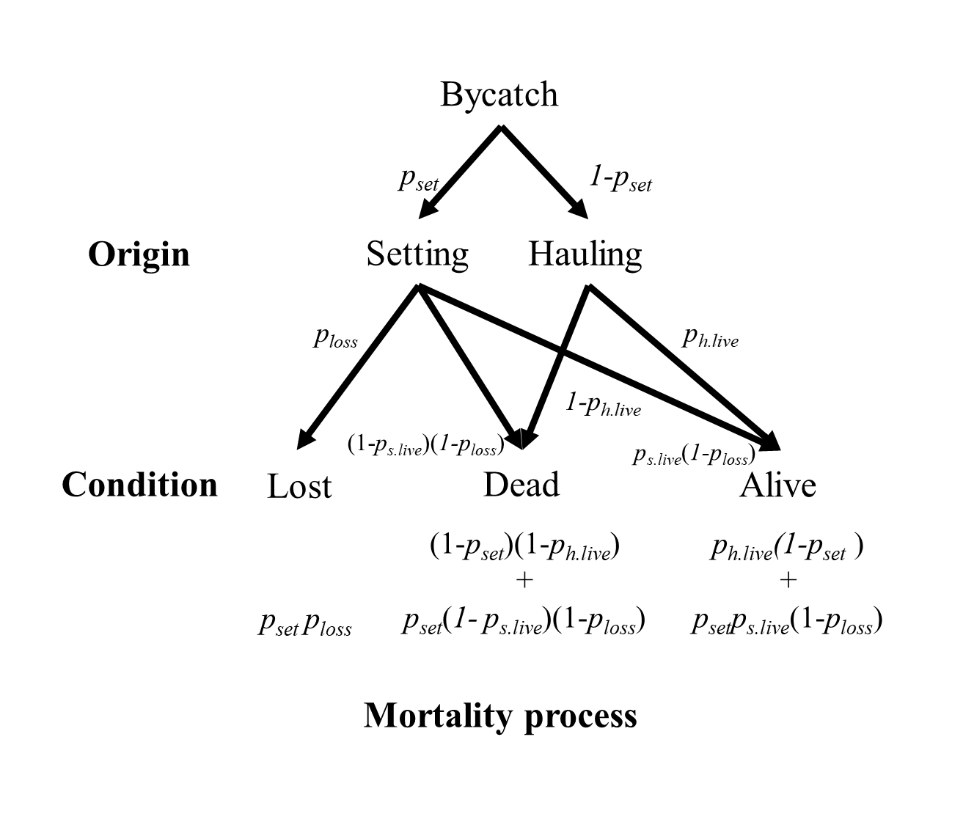


S1 Figure. A general mortality process. In addition to a positive survival rate for the haul-captures (*p_h.live_*), the survival rate for set-captures may also be positive (*p_s.live_*). When *p_s.live_* is set to zero, the probabilities coincide with those in Fig 3.

## References

1. Gilman E, Chaloupka M, Peschon J, Ellgen S. Risk Factors for Seabird Bycatch in a Pelagic Longline Tuna Fishery. PloS one. 2016;11(5):e0155477.
